# Replacing protein with carbohydrate or fat in infancy is associated with lower Body Mass Index in early childhood: results from the Melbourne InFANT Program

**DOI:** 10.1038/s41366-026-02099-y

**Published:** 2026-05-07

**Authors:** Tinsae Shemelise Tesfaye, Ewa A. Szymlek-Gay, Carley A. Grimes, Miaobing Zheng

**Affiliations:** 1 Institute for Physical Activity and Nutrition (IPAN), School of Exercise and Nutrition Sciences, Deakin University, Melbourne, VIC Australia; 2 School of Health Sciences, Faculty of Health & Medicine, University of New South Wales, Sydney, NSW Australia

**Keywords:** Obesity, Risk factors

## Abstract

**Background:**

Higher intakes of total and animal-source protein during infancy have been associated with higher body mass index (BMI) z scores in childhood.

**Objective:**

We examined the association of substituting protein with fat or carbohydrate intake, and the substitution of protein subtypes at 9 months of age with BMI z-scores and overweight status in children at 5 years of age.

**Methods:**

This study involved a secondary analysis of data from the Melbourne InFANT program, a 15-month infancy obesity prevention intervention, with additional follow-ups conducted without intervention when children were aged 3.5 and 5 years. Data of 345 children who completed the 9-month, and 5-year follow-ups were analysed. Dietary intake at 9 months was assessed using three 24-h recalls. BMI z-score was measured at 9 months and 5 years of age. Multivariable linear and logistic regression models with adjustment for potential confounders examined the associations between macronutrient and protein subtype substitutions at 9 months and changes in BMI z-score or overweight status at 5 years.

**Results:**

Substitution of 5%E or 100 kJ protein intake with carbohydrate intake at age 9 months was associated with a 0.16-unit (95% CI: −0.30, −0.02) or 0.11-unit (95% CI: −0.19, −0.03) decrease in BMI z-score at 5 years. Similarly, replacing 5%E or 100 kJ of protein intake with fat intake was associated with a 0.16-unit (95% CI: −0.29, −0.02) or 0.11-unit (95% CI: −0.19, −0.03) decrease in BMI z-score. There was no evidence of an association between substitution of protein with fat or carbohydrate intake and overweight status (*P* > 0.05). Replacement of animal protein with plant or dairy protein was also not significantly associated with BMI z-score or overweight status.

**Conclusions:**

Substituting protein with carbohydrate or fat intake in infancy was inversely associated with BMI z-score in early childhood. The present study supports the need to discourage excessive protein intake during infancy.

## Introduction

Childhood obesity has been one of the most critical global health challenges for decades [1]. In 2022, the World Health Organization (WHO) estimated that 37 million children under the age of 5 years were living with overweight worldwide [2]. In Australia, a quarter of children and adolescents aged 2–17 years were living with overweight or obesity in 2017–18 [3]. A large body of evidence indicates that childhood obesity persists into adulthood and is associated with adverse short- and long-term health and socioeconomic consequences [4, 5]. The Developmental Origins of Health and Disease (DOHaD) hypothesis highlights that the first 1000 days of a child’s life, from preconception to age 2 years, constitute a crucial period for growth and development with a long-lasting impact on health outcomes, including obesity [6].

Nutrition in early life has a significant and enduring effect on the development of childhood overweight and obesity [7, 8]. For example, macronutrient intakes, particularly excessive protein intake in infancy, have been associated with an increased risk of childhood obesity [9, 10]. The rapid increase in protein intake during the period of complementary feeding and the transition to the family diet from 6 months to 1 year of age is particularly crucial [11, 12]. Moreover, accumulating evidence suggests that protein intake from non-dairy animal sources, but not plant sources, in infancy is associated with higher weight gain in childhood [9, 10]. Findings for dairy protein remain inconsistent [13, 14]. In contrast, carbohydrate and fat intake in infancy have not been associated with subsequent body weight development [15, 16]. Whether reducing protein intake in infancy by replacement of fat or carbohydrate may influence body weight development remains unclear. Such information might be valuable for informing dietary guidelines on macronutrient intake and intervention strategies to support optimal macronutrient intakes in infancy.

Emerging studies have explored the simulated effect of macronutrient intake substitution on body weight outcomes using substitution modelling, which assesses the association between isocaloric substitution of dietary components and health outcomes. For example, estimating the simulated effect of replacing one macronutrient with another on body weight outcomes, while keeping total energy constant [17, 18]. The majority of macronutrient substitution studies have been conducted in adults [19, 20] and school-aged children [21, 22], and very few in early childhood. However, no studies to date have assessed the association between substituting protein with other macronutrients (i.e., carbohydrate or fat), or the substitution of protein subtypes, before 1 year of age and later obesity outcomes. Research has shown a differential association between protein intake and body weight outcomes across the lifespan. In contrast to the detrimental role of high protein intake in infancy on later obesity risk [9, 10], high protein intake in adolescence and adulthood has been found to protect against obesity [23, 24]. These contrasting associations across life stages highlight the need to assess macronutrient substitution during infancy and association with body weight outcomes in childhood. Therefore, this study aimed to examine the associations of substituting protein intake with fat or carbohydrate intake, as well as the substitution of different protein subtypes (e.g., non-dairy animal, plant, and dairy) at age 9 months and body weight outcomes at 5 years.

## Methods

### Study population

This study is a secondary analysis of data from the Melbourne Infant Feeding Activity and Nutrition Trial (InFANT) Program, a 15-month parent-focused intervention trial designed to assess the effectiveness of an early childhood obesity prevention program (Clinical Trial registration: Current controlled Trials ISRCTN81847050). A total of 542 parent-child pairs from 62 first-time parent groups were included at baseline when infants were around 4 months of age. The intervention was delivered to first-time parents to promote healthy eating and active play in infants from 4 to 18 months of age [25]. The intervention group received educational sessions, while the control group received standard care from June 2008 to February 2010 [25, 26]. Additional follow-ups without intervention were conducted to investigate the sustainability of the intervention effects when the children were 3.5 and 5 years old (2011–2013) [27]. Detailed trial protocol and outcome evaluation results were reported previously [25–27]. Ethical approval for the Melbourne InFANT program was obtained from the Deakin University Human Research Ethics Committee (ID no.: EC 175-2007) and the Victorian Government, Office for Children (ref: CDF/07/1138). All participants provided written informed consent.

The current analyses included data from children aged 9 months and 5 years. In line with previous analyses [13, 28], data were excluded for children from non-first-time mothers (*n* = 14) and those with fewer than 3 days of dietary recalls or outlier total energy intake (±3 standard deviations) [13, 28]. This resulted in a sample of 480 children with complete data of 3-day dietary recalls and BMI at age 9 months, of whom 345 completed anthropometric measurements at age 5 years (Supplementary Fig. S1).

### Dietary assessment

Children’s dietary intake was assessed at the age 9 months using standardized telephone-administered multiple-pass 24-h recalls. Trained nutritionists interviewed parents of children by telephone on three non-consecutive days, including two weekdays and one weekend day [25, 27]. The majority (96%) of the dietary recalls were unscheduled to minimize response bias. For children who spent time with multiple caregivers’, food diaries were completed by other carers (e.g., daycare provider or grandparent) during child-care periods on scheduled days. These were then used by parents to provide detailed 24-h recalls (4% of the total recalls) [25, 27].

Specially designed booklets containing photographs of standard portion sizes and examples of measurements, such as cups, bowls, drink containers, and spoons, were provided to aid in estimating the food portion sizes [25, 27]. Consistent with previous studies [13, 29], breast milk intake was assessed as minutes spent breastfeeding and converted into volumes using a conversion factor of 10 mL/min up to a maximum of 100 mL for any feed. If breast milk was expressed, volumes estimated by caregiver reports were used [13]. The 2007 Australian Food, Supplement, and Nutrient Food Composition Database (AUSNUT 2007) [30] was used to derive total energy and nutrient intake from breast milk, infant formula, foods, and beverages. Daily energy intake (kJ/d), total protein, carbohydrate, and fat intakes (g/d) were calculated. The energy derived from protein, carbohydrates, and fat was calculated using standard energy conversion factors from grams to kilojoules: 17 kJ/g for carbohydrate and protein and 37 kJ/g for fat [31]. The percentage of energy (%E) from each macronutrient was determined by dividing the energy provided by each macronutrient by the total energy intake, expressed as a percentage [31]. Total protein intake was categorized into 3 major sources and calculated in grams per day and %E: dairy animal protein (derived from dairy foods such as infant formula; breast milk; and cow’s, sheep’s, or goat’s milk, yogurt, and cheese), non-dairy animal protein (derived from animal-based foods such as eggs, fish, poultry, and red meat), and plant protein (derived from plant-based foods such as cereals, legumes, vegetables, and fruits). Further, children’s primary milk source was classified as breastmilk, formula/dairy, or mixed following the approach used in previous InFANT studies [29]. A milk source was considered primary if it contributed more than two-thirds (66.7%) of total milk intake; otherwise, the source was classified as mixed. Only 5 children primarily consumed dairy milk at 9 months, so they were combined with the formula group due to their protein content being more similar to formula than to breastmilk. All analyses were based on the mean daily dietary intake over 3 days.

### Child anthropometric characteristics

At ages 9 months and 5 years, trained research staff measured children’s length/height and weight [13, 25]. At each timepoint, two measurements of length and height were taken, and the mean of the two measurements was used in analysis. Children’s weight was measured to the nearest 10 g using calibrated digital scales (Tanita 1582; Tanita Corp.) while wearing light clothes and without shoes. Length/height was measured bare feet twice to the nearest 1 mm using a mobile measuring mat at 9 months of age (Seca 210, Seca Deutschland) and a portable stadiometer (Invicta IPO955; Oadby) at 5 years of age. The World Health Organization’s age- and sex-specific growth charts were used to compute BMI z-scores [32]. Overweight and obesity were defined according to the WHO definition [2]: overweight as a BMI z-score >1 and ≤2 for children aged 5 years, and obesity as a BMI z-score >2 for children aged 5 years.

### Child and maternal sociodemographic characteristics

A range of child and maternal characteristics were collected at the study baseline (4 months), including the child’s sex (boy vs girl), gestational age in weeks, maternal age in years, maternal country of birth (Australia vs other), maternal educational level, maternal height, maternal pre-pregnancy body weight, and maternal age at the birth of the child using self-administered questionnaires. Covariates and potential moderators were selected based on a directed acyclic graph (Supplementary Fig. S2), which was constructed based on theory and the findings of prior literature. Maternal education was dichotomized as below university (no formal education, high school, trade/apprenticeship/certificate/diploma) or university or higher (university degree or higher). Mothers provided self-reported data on their pre-pregnancy weight and height, and Body Mass Index (BMI) (kg/m^2^) was calculated as weight in kg divided by height in meters squared (m^2^). Information on child birthweight was reported by mothers from their children’s birth records [26, 27].

### Statistical analysis

Comparisons of the mean daily protein, fat, and carbohydrate intake (g/d), and BMI z-scores between the intervention and control groups at ages 9 months and 5 years were conducted to identify differences between the two groups. As the results showed no statistical differences (Supplementary Table S1), data from the intervention and control groups were pooled for the current analysis. In addition, intervention allocation was included as a covariate in the analyses to account for potential confounding due to intervention allocation. Differences in protein, carbohydrate, and fat intake (g/d), BMI z-scores, and overweight status (yes vs no) by baseline characteristics were assessed by *t*-test and chi-square test, respectively. Mixed-effects multivariable linear and logistic regression models, specifying parent groups as random effects to account for clustering, were used to examine associations between macronutrient substitution at ages 9 months and changes in BMI z-score or overweight status at ages 5 years, respectively. The leave-one-out macronutrient model with regression coefficients directly providing substitution effects [33] was used for the substitution analysis. This model included total energy intake and intake of each macronutrient (fat and carbohydrate) but excluded protein intake as a reference category. This method allows the evaluation of the simulated impact of substituting protein with fat or carbohydrate on BMI z-score and overweight status. In line with previous studies [33, 34], the substitution model in this study was performed considering macronutrient intake in %E (per 5%E increase) or kilojoules (per 100 kJ increase) from each macronutrient.

$$\mathrm{\varDelta BMIz}-\mathrm{score}={\rm{\beta }}{1}^{* }\mathrm{Carbohydrate}+{\rm{\beta }}{2}^{* }\mathrm{fat}+{\rm{\beta }}{3}^{* }\mathrm{total\; energy}$$

In the substitution model, β1 to β3 represent regression coefficients. For example, the coefficient of carbohydrate (β1) can be interpreted as the estimated change in BMI z-score by a 5%E or 100 kJ increase in carbohydrate at the expense of the same amount of protein while keeping total energy and fat intakes constant. If total energy and fat intake are held constant, an increase in carbohydrate intake results in a corresponding decrease in protein intake. Similarly, the coefficient of fat has the same substitution meaning [33, 34]. We conducted both crude and adjusted models. The crude models were adjusted for intervention allocation and BMI z-score at age 9 months (Model 1). Adjusted models, informed by the directed acyclic graph, included child sex (boys vs girls), children’s primary milk source at 9 months (breastmilk, formula/dairy, or mixed), birthweight, maternal education (below university vs university or higher), and maternal pre-pregnancy BMI (kg/m²) (Model 2). The simulated effects of replacing non-dairy animal protein with plant or dairy protein were assessed in the same manner. The model includes total amount of protein intake, plant and dairy protein while excluding non-dairy animal protein from the model [33, 34].

$$\mathrm{\varDelta BMI\; z}-\mathrm{score}={\rm{\beta }}{1}^{* }\mathrm{plant\; protein}+{\rm{\beta }}{2}^{* }\mathrm{dairy\; protein}+{\rm{\beta }}{3}^{* }\mathrm{total\; protein}$$

The coefficient for plant protein (β1) represents the effect of substituting 5%E or 5 g non-dairy animal protein with an equal amount of plant protein on the BMI z-score while holding total protein and dairy protein constant. Similarly, the coefficient of dairy protein has the same substitution meaning. The crude model was adjusted for intervention allocation and BMI z-score at age 9 months (Model 1). Further, the models additionally adjusted for the same covariates as the model above. To evaluate whether total energy intake has a confounding effect on the association, additional adjustment for total energy intake at age 9 months was performed (Model 3). Model assumptions for regression analysis were checked using standard diagnostic plots, and all assumptions were met. All statistical analyses were performed using STATA version 18, and two-sided *p*-value < 0.05 were considered statistically significant.

### Sensitivity analysis

Stratified analyses were conducted to test whether the association between substituting protein with fat or carbohydrate intake, and BMI z-scores or overweight status differed by intervention group. Additionally, potential interactions between the macronutrient substitution models and intervention allocation were assessed by including interaction terms in the adjusted models.

## Results

Comparisons of total protein, carbohydrate, and fat intake (g/d) at age 9 months by child and maternal characteristics are shown in Table 1. Mean ± SD total protein, carbohydrate, and fat intakes (g/d) at age 9 months were 28.2 ± 11.0, 98.9 ± 26.1, and 33.7 ± 8.2, respectively (Supplementary Table S2). Total protein intake comprised 50% dairy protein, 22% non-dairy animal protein, and 28% plant protein At 5 years of age, the children had a mean height, weight, and BMI z-score of 111.0 ± 4.9 cm, 19.9 ± 2.7 kg, and 0.5 ± 0.9, respectively. Of 345 children included in the study, 64 (18.6%) were classified as having overweight and 13 (3.8%) as having obesity at age 5 years (Supplementary Table S3).

**Table 1:** Comparison of mean ± SD of total protein, carbohydrate, and fat intake (g/d) by child and maternal characteristics of children (*n* = 345) who participated in both 9-month and 5-year examinations in the Melbourne Infant Feeding Activity and Nutrition Trial program.

	Overall mean ± SD/*n* (%)	Mean ± SD of total protein	*P*-value^a^	Mean ± SD of CHO	*P*-value	Mean ± SD of fat	*P*-value
Group							
Control	171 (49.6%)	29.1 (11.1)	0.62	98.1 (23.6)	0.57	33.7 (8.0)	0.99
Intervention	174 (50.4%)	29.7 (11.2)		99.7 (28.3)		33.6 (8.4)	
Child sex							
Boys	183 (53.0%)	31.2 (11.7)	0.002	105.1 (27.0)	<0.001	32.3 (8.5)	0.004
Girls	162 (47.0%)	27.4 (9.9)		91.9 (23.1)		32.3 (7.7)	
Primary milk source							
Breastmilk	125 (36.2%)	22.7 (9.1)	0.21	82.1 (23.7)	0.71	30.5 (7.9)	0.99
Formula/dairy	194 (56.2%)	33.3 (10.5)		109.1 (22.4)		35.5 (7.9)	
Mixed	26 (7.5%)	33.0 (9.6)		103.7 (21.5)		35.5 (7.7)	
Gestational age							
Pre-term (<37 wk)	40 (11.6%)	32.2 (10.3)	0.09	106.0 (25.1)	0.07	35.1 (7.7)	0.22
Full-term (≥37 wk)	304 (88.4%)	29.1 (11.1)		98.0 (26.1)		33.4 (8.2)	
Birthweight							
Low (<2.5 kg)	24 (7.0%)	34.4 (11.3)	0.03	108.7 (26.4)	0.06	36.5 (8.4)	0.08
Normal (≥2.5 kg)	318 (93.0%)	29.1 (11.1)		98.2 (26.0)		33.4 (8.2)	
Country of birth							
Australia	273 (81.7%)	29.9 (11.3)	0.09	100.1 (26.8)	0.05	33.8 (8.2)	0.53
Other	61 (18.3%)	27.3 (10.3)		92.8 (22.2)		33.0 (8.2)	
Educational level							
University or higher	209 (60.6%)	28.2 (9.9)	0.02	95. 4 (23.5)	0.002	33.2 (7.5)	0.19
Below university	136 (39.4%)	31.2 (12.5)		104.3 (28.8)		34.4 (9.1)	
Maternal age at baseline, years	32.4 ± 4.1		0.71		0.10		0.34
Maternal pre-pregnancy BMI	24.2 ± 5.3		0.10		0.18		0.20

*CHO* carbohydrate, *BMI* body mass index, calculated as kg/m^2^.

^a^Differences between total protein, carbohydrate, and fat (g/d) intake and overweight status were assessed using the *t*-test.

Table 2 presents the regression analysis results examining the associations between substituting protein intake with carbohydrate or fat intake at age 9 months and BMI z-score or overweight status at age 5 years. After adjusting for covariates, substitution of protein intake (5%E) with carbohydrate intake was associated with a 0.16-unit decrease in BMI z-score at 5 years of age (β = −0.16; 95% CI: −0.30, −0.02; *P* = 0.03). Similarly, replacing 5%E of protein intake with fat intake was associated with a 0.16-unit decrease in BMI z-score (β = −0.16; 95% CI: −0.29, −0.02; *P* = 0.02) (Model 2). Likewise, replacing every 100 kJ of protein intake with either carbohydrate or fat intake was associated with a 0.11-unit decrease in BMI z-score at 5 years (carbohydrate: β = −0.11; 95% CI: −0.19, −0.03; *P* = 0.01; fat: β = −0.11; 95% CI: −0.19, −0.03; *P* = 0.01). No statistically significant association was observed between substituting protein intake (5%E or per 100 kJ) with an equivalent amount of fat or carbohydrate and lower odds of being overweight at age 5 years, but a consistent inverse trend was found (Table 2, Model 2).

**Table 2:** Regression analysis results for effects of substituting carbohydrate and fat intake for protein intake on changes in BMI z-score and overweight status of children from ages 9 months to 5 years.

	Substituting protein (5%E)					
	BMI z-score			Overweight status		
	β	95% CI	*P*-value	OR	95% CI	*P*-value
Model 1 (*n* = 345)^a^						
Carbohydrate	−0.15	−0.29, −0.003	0.05	0.75	0.45, 1.26	0.28
Fat	−0.13	−0.26, −0.01	0.07	0.73	0.44, 1.21	0.22
Model 2 (*n* = 331)^b^						
Carbohydrate	−0.16	−0.30, −0.02	0.03	0.70	0.41, 1.20	0.20
Fat	−0.16	−0.29, −0.02	0.02	0.63	0.37, 1.07	0.09
	**Substituting protein (100** **kJ)**					
	**BMI z-score**			**Overweight status**		
	**β**	**95% CI**	***P*-value**	**OR**	**95% CI**	***P-*value**
Model 1 (*n* = 331)						
Carbohydrate	−0.10	−0.19, −0.02	0.01	0.85	0.64, 1.14	0.29
Fat	−0.10	0.18, −0.01	0.03	0.86	0.63, 1.17	0.33
Model 2 (*n* = 331)						
Carbohydrate	−0.11	−0.19, −0.03	0.01	0.84	0.62, 1.14	0.27
Fat	−0.11	−0.19, −0.03	0.01	0.81	0.59, 1.12	0.21

The models represent the iso-energetic increase of 5%E, or a 100 kJ increase from one macronutrient (carbohydrate or fat) with a decrease in the substituting macronutrient (protein).

^a^Model 1 was adjusted for intervention allocation, and body mass index (BMI) z-score at 9 months of age.

^b^Model 2 was additionally adjusted for child sex, primary milk source (breastmilk, formula/dairy, or mixed), child birthweight, maternal education, and pre-pregnancy BMI.

Table 3 presents the association between the substitution of non-dairy animal protein with plant or dairy protein at 9 months of age and BMI z-score and overweight status at 5 years of age. Substituting non-dairy animal protein intake (5%E, or 5 g) with an equivalent amount of plant or dairy protein at 9 months was not associated with BMI z-score or overweight status at 5 years of age (*P* > 0.05) (Fully adjusted model, model 3).

**Table 3:** Regression analysis results for effects of substituting plant and dairy protein for non-dairy animal protein on changes in BMI z-score and overweight status from 9 months to 5 years of age.

	Substituting non-dairy animal protein (5%E)					
	BMI z-score			Overweight status		
	β	95% CI	*P*-value	OR	95% CI	*P*-value
Model 1 (*n* = 345)^a^						
Plant protein	0.003	−0.28, 0.29	0.99	1.42	0.53, 3.81	0.48
Dairy protein	−0.12	−0.35, 0.11	0.31	0.91	0.41, 2.06	0.83
Model 2 (*n* = 331)^b^						
Plant protein	0.03	−0.24, 0.31	0.82	1.52	0.53, 4.36	0.44
Dairy protein	−0.02	−0.28, 0.25	0.91	1.22	0.44, 3.38	0.71
Model 3 (*n* = 331)^c^						
Plant protein	0.05	−0.23, 0.33	0.72	1.34	0.46, 3.92	0.60
Dairy protein	−0.02	−0.29, 0.24	0.87	1.22	0.44, 3.41	0.70
**Substituting non-dairy animal protein (5 g)**						
	**BMI z-score**			**Overweight status**		
	**β**	**95% CI**	***P*-value**	**OR**	**95% CI**	***P*-value**
Model 1 (*n* = 345)						
Plant protein	−0.02	−0.13, 0.09	0.75	1.08	0.75, 1.57	0.68
Dairy protein	−0.07	−0.16, 0.02	0.15	0.94	0.68, 1.29	0.69
Model 2 (*n* = 331)						
Plant protein	−0.003	−0.11, 0.11	0.96	1.12	0.75, 1.68	0.58
Dairy protein	−0.04	−0.15, 0.71	0.49	1.10	0.73, 1.68	0.63
Model 3 (*n* = 331)						
Plant protein	0.06	−0.06, 0.18	0.36	1.11	0.70, 1.74	0.66
Dairy protein	−0.02	−0.10, 0.15	0.72	1.09	0.68, 1.75	0.72

The models represent the iso-energetic increase of 5%E, or 5 g increase from one macronutrient subtype (plant or dairy protein) with a decrease in the substituting macronutrient (non-dairy animal protein).

^a^Model 1 were adjusted for intervention allocation, and body mass index (BMI) z-score at 9 months of age.

^b^Model 2 was additionally adjusted for child sex, primary milk source (breastmilk, formula/dairy, or mixed), child birthweight, maternal education, and pre-pregnancy BMI.

^c^Model 3 was additionally adjusted for total energy intake at 9 months.

### Sensitivity analysis

Substituting protein with carbohydrate or fat was significantly associated with lower BMI z-scores in the control group but not in the intervention group; however, the direction of associations was consistent across both groups (Supplementary Table 4). No significant differences were observed between intervention groups for overweight status (Supplementary Table 4), nor for substitutions involving different protein sources and BMI z-score or overweight status (Supplementary Table 5). Moreover, no significant interactions were detected between intervention allocation and carbohydrate and fat intakes in substitution models. The likelihood ratio test revealed no significant difference in stratum-specific effect sizes between intervention and control groups (*P* > 0.05), indicating that the simulated effects of macronutrient substitution on BMI z-scores did not differ by intervention allocation.

## Discussion

This is the first study to investigate the association between protein intake substitution in infancy and body weight outcomes in young Australian children. Substituting protein intake with fat or carbohydrate intake at 9 months of age was associated with a lower BMI z-score, but not overweight status at age 5 years. Substituting animal protein with plant or dairy protein was not associated with BMI z-score or overweight status.

Our findings that replacing protein with fat or carbohydrates in infancy was associated with lower BMI z-score in early childhood are consistent with two previous studies in European children. For example, in a large cohort of Dutch children (*n* = 3573), substituting protein with carbohydrate intake at 1 year of age was associated with lower BMI and fat mass index at age 10 years [16]. Similarly, a study conducted in a large sample of UK children (*n* = 2154) demonstrated that replacing protein with either carbohydrates or fat at age 21 months was linked to decreased weight and BMI at age 5 years [35]. Furthermore, the UK study also reported no significant association between replacing protein with carbohydrate or fat and the odds of being overweight at age 5 years [35]. A possible explanation for the lack of significant association between protein intake substitution and overweight status in our study may be attributable to limited statistical power as a result of small sample size and the relatively low prevalence of overweight (18.6%). Likewise, the UK study also reported that the low prevalence of overweight (6%) in their sample may have contributed to the null findings [35]. It is worth noting that, in the current study, the inverse association between replacing protein with carbohydrate or fat and BMI z-score was evident only in the control group. However, the direction of the associations was consistent across groups, and interaction analyses showed no evidence of significant effect modification. These apparent differences between intervention groups likely reflect reduced subgroup sample sizes rather than true differences in effect. The significant association in the control group indicates that the substitution association is irrespective of the intervention.

Several explanations may support the benefits of replacing protein with fat or carbohydrates during infancy in reducing risk of obesity in early childhood. Reducing protein intake, while increasing fat or carbohydrate intake, might alleviate these harmful effects of high protein intake in infancy [36]. It has been proposed that excess protein intake during infancy may increase the secretion of insulin and insulin-like growth factor I [9, 10], which can accelerate body weight gain and increase the long-term risk of obesity. Additionally, infants with low protein intake tend to be breastfed, and those with high protein intake tend to be formula-fed [29]. Current evidence on the impact of breastfeeding on childhood overweight and obesity is equivocal; however, mounting evidence supports its beneficial role in reducing obesity risk. [37, 38]. Previous studies suggest that breastfed infants tend to gain weight slowly than non-breastfed infants [37, 39], which may reduce the risk of obesity later in childhood. The composition of breastmilk, such as lower protein content and the presence of bioactive factors, including appetite-regulating hormones like leptin and ghrelin, may also play a role in protecting against obesity [37, 38]. Moreover, diets high in fat and carbohydrate in infancy, such as whole-milk consumption may enhance satiety and reduce overall energy intake, thereby lowering the risk of excessive weight gain [40]. Conversely, restricting fat intake in early life may trigger adaptive metabolic responses that favour energy storage, increasing susceptibility to later overweight, metabolic disorders, and leptin resistance [8]. Further, current evidence showed either no deleterious effect of high consumption of fat or carbohydrate during the first two years of life, or even protective effects of higher fat in early childhood [8, 15]. Thus, moderating protein intake in infancy and exploring the potential benefits of higher carbohydrate or fat intake might constitute a potentially important approach for reducing the risk of childhood overweight.

We are also the first study to examine the association between the substitution of protein subtypes in infancy and obesity outcomes in early childhood. Despite previous evidence revealed an association of non-dairy animal protein in infancy and increased obesity risk [13, 41], we found no beneficial association of reducing non-dairy animal protein by replacing it with either plant protein or dairy protein and obesity outcomes. The lack of a significant association in our study may be explained by the low intake of plant protein and the relatively small sample size, which may have limited variability and statistical power. Additionally, unmeasured or residual confounding from other dietary or lifestyle factors could have also influenced the observed associations. Past research examined substitution of protein subtypes in older children [21] and adults [19] has shown a beneficial impact of replacing animal protein with plant protein on obesity risk. However, these studies are not directly comparable to ours due to differences in age, study design, protein sources classification (animal vs plant or non-dairy animal vs dairy vs plant), and outcome measures (BMI z-scores vs body fat or composition). Further research is needed to examine the impact of protein and protein subtype substitution in infancy on obesity outcomes.

The current study has several strengths. Apart from being the first to investigate the association between protein and protein subtype substitutions early in infancy, our study collected high-quality dietary data via three 24-h recalls over three non-consecutive days. Furthermore, trained staff conducted objective anthropometric measurements, unlike some studies, which relied on self-reported data [35]. The present study has some limitations that warrant discussion. The small sample size may have limited the statistical power to detect significant associations, such as protein substitution and overweight status. Given the observational nature of our study, unmeasured and residual confounding from other obesity contributing factors, such as physical activity [42], sedentary behaviour [43], and sleep duration [44] is possible. The use of a single measure of dietary intake at one time point (9 months) without considering changes in diet over time is another limitation. Additionally, BMI z-score does not account for body composition (lean mass vs body fat). Further research will be desirable to assess the association between protein intake substitution in infancy and body composition. Another limitation of our study is the absence of blood or other biochemical samples to objectively assess macronutrient intake. Dietary intake information was based on parent reports; therefore, reporting errors and recall bias cannot be ruled out. Additionally, breastmilk intake may have been under- or overestimated, as the volume assumption did not account for individual differences in feeding efficiency, milk flow rate, or infant body weight [45]. As a result, some degree of error in total energy intake estimation is possible. Future studies should incorporate dietary biomarkers to improve the accuracy of nutrient and energy intake assessments in infants and young children. Finally, the overrepresentation of university-educated women may limit the generalizability of the study findings to the broader Australian population.

Extending findings from previous studies that showed harmful role of high protein intake in infancy in later obesity development [9], our study highlights the potential benefits of balancing protein intake with carbohydrate and fat in infancy for prevention of early childhood obesity. However, it is important to acknowledge that protein is essential for healthy growth and development, and many protein-rich food sources also provide important nutrients. Future research should determine the optimal protein intake required for healthy growth and body composition in early childhood.

## Conclusion

Our findings suggest that substituting protein with carbohydrate or fat intake in infancy was associated with BMI z-score, but not overweight status in early childhood. No evidence of an association was found between replacing non-dairy animal protein with plant or dairy protein in infancy and obesity outcomes in early childhood. This information will be valuable for informing the refinement of macronutrient intake recommendations during infancy and infant feeding guidelines. However, further rigorously designed studies in diverse populations that incorporate comprehensive adiposity measures (e.g., body composition) are needed to better understand the long-term impact of protein intake and protein subtype substitutions on the development of obesity later in life.

## Supplementary information

Supplementary material

## Data Availability

The data are not publicly available as they contain information that could compromise research participant privacy. However, deidentified data are available upon request with a methodologically sound proposal via gavin.abbott@deakin.edu.au and pending approval from the relevant ethics committee

## Code availability

Code for all published analyses are available upon written request.
