# Supplementary material for: Replacing protein with carbohydrate or fat in infancy is associated with lower Body Mass Index in early childhood: results from the Melbourne InFANT Program
Source: Int J Obes (Lond). 2026 May 7;50(7):1566–72. doi: 10.1038/s41366-026-02099-y (PMC13391387; doi:10.1038/s41366-026-02099-y)
Supplement: Supplementary file 1 — Supplementary material [file 41366_2026_2099_MOESM1_ESM.pdf]

Replacing protein with carbohydrate or fat in infancy is associated with lower Body Mass Index in early childhood

Tinsae Shemelise Tesfaye[1], Ewa A. Szymlek-Gay[1], Carley A. Grimes[1], Miaobing Zheng[1,2]

[1] Institute for Physical Activity and Nutrition (IPAN), School of Exercise and Nutrition Sciences, Deakin University, Melbourne, VIC, Australia

[2] School of Health Sciences, Faculty of Health & Medicine, University of New South Wales, Sydney, NSW, Australia

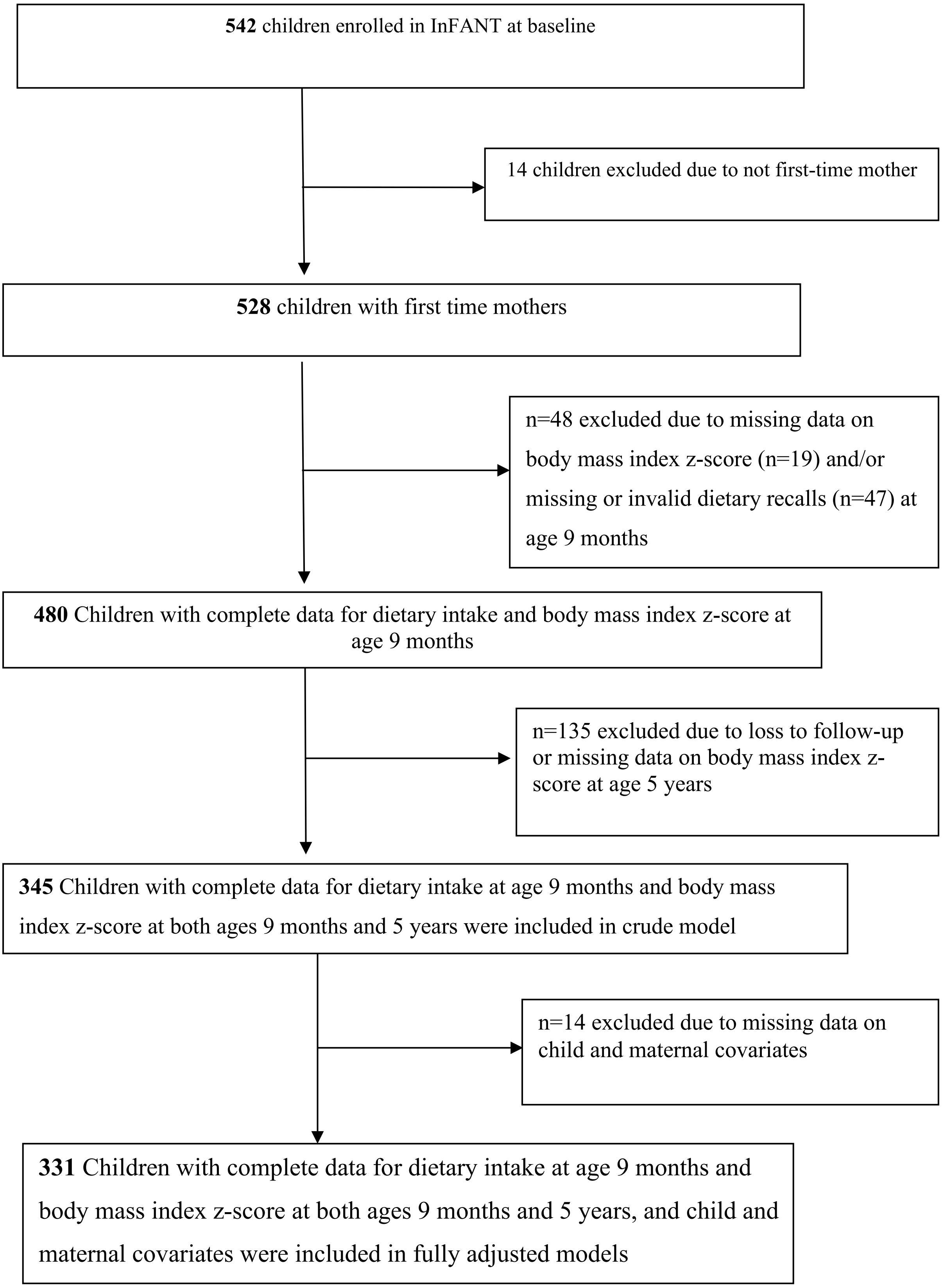

Supplementary Figure 1: Flow chart showing the number of participants included in the analysis of associations between macronutrient intake substitution and body mass index (BMI) z-score in early childhood.

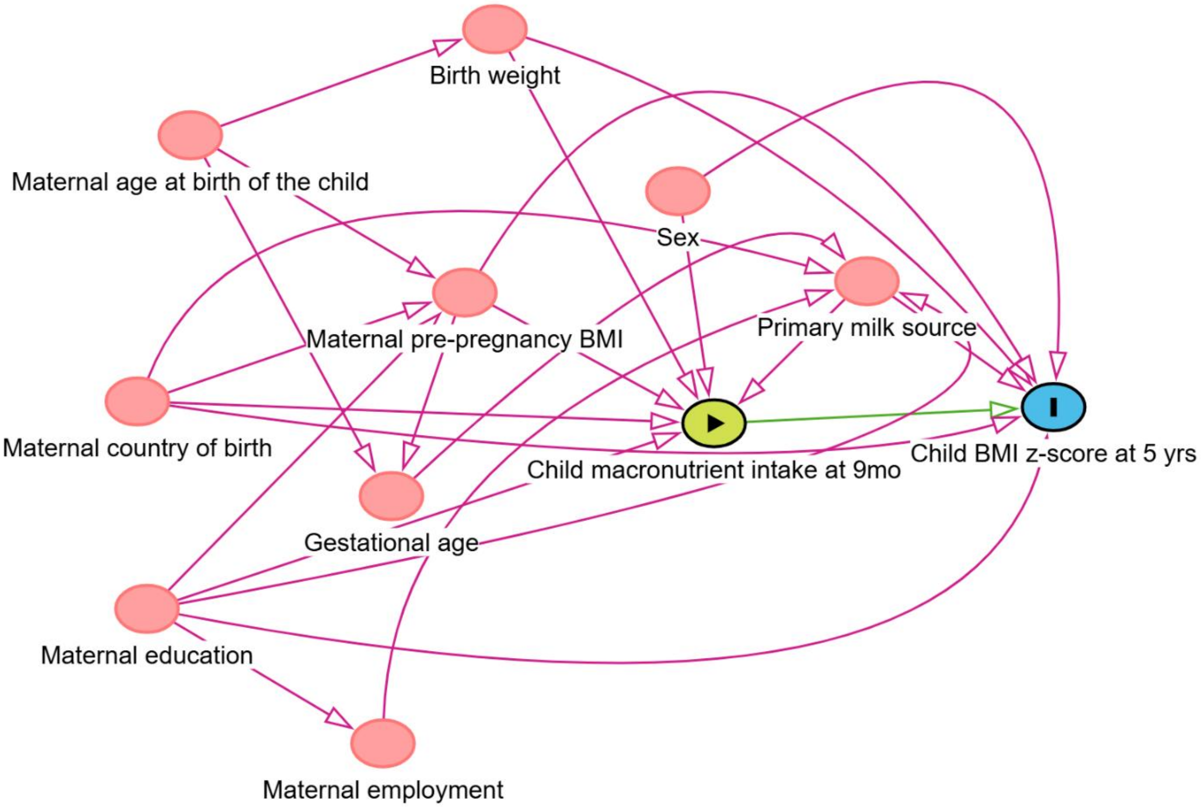

Supplementary Figure 2 Directed Acyclic Graph for the association between macronutrient intake substitution and body mass index (BMI) z-score in early childhood. Note: Primary milk sources (breastmilk, formula/dairy, or mixed).

Supplementary Table S1 Comparison of the mean ± SD total protein, carbohydrate, and fat intake (g/d) and BMI z-score at 9 months, and 5 years by treatment group in the Melbourne Infant Feeding Activity and Nutrition Trial (InFANT) Program.

**Time point**	**Treatment group**	**n**	**Mean ± SD of total protein, g/d**		**P-value[a]**	**Mean ± SD of fat, g/d**		**P-value[a]**	**Mean ± SD of CHO, g/d**		**P-value[a]**	**Mean ± SD of BMI z-scores**		**P-value[a]**
9 months	Control	171	29.1	11.1	0.62	33.7	8.0	0.98	98.1	23.6	0.57	0.09	1.03	0.73
	Intervention	174	29.7	11.2		33.6	8.4		99.7	28.3		0.13	0.94	
	Control	129	60.4	15.0	0.95	49.5	14.9	0.7	176.1	36.3	0.69	0.53	0.93	0.82
5 years	Intervention	129	60.6	14.9		48.8	16.1		174.3	39.3		0.55	0.93	

[a] Differences between macronutrient intake and BMI z-score were assessed by t test, CHO: carbohydrate.

Supplementary Table S2 Energy and macronutrient intakes at ages 9 months in Melbourne Infant Feeding Activity and Nutrition Trial (InFANT) Program (n =345).

	Mean ± SD	
Energy intake (kJ/d)	3478 ± 841	
	g/d	%E
Total protein intake	28.2 ± 11.0	13.5 ± 3.0
Protein subtypes		
Non-dairy animal protein	7.1 ±7.4	3.3 ±3.1
Dairy protein	13.4 ±5.6	6.5±2.1
Plant protein	7.7 ±4.7	3.7 ±1.7
Fat intake	33.7 ± 8.2	36.2 ±5.4
Carbohydrate intake	98.9 ± 26.1	48.3 ± 4.8

Abbreviation: SD standard deviation, %E percent total energy intake.

Supplementary Table S3. Anthropometric characteristics of children at age 5 years in Melbourne Infant Feeding Activity and Nutrition Trial (InFANT) Program (n =345).

Characteristic	Mean ± SD/ n (%)
Height, cm	111.0 ± 4.9
Weight, kg	19.9 ± 2.7
BMI z-score	0.5 ± 0.9
Children with normal weight, n (%)	268 (77.7)
Children with overweight, n (%)	64 (18.6)
Children with obesity, n (%)	13 (3.8)

Abbreviation: BMI: body mass index, calculated as kg/m2

Supplementary Table S4 Stratified analysis results by intervention allocation for effects of substituting carbohydrate and fat for protein on changes in BMI z-score and overweight status from 9 months to 5 years of age.

	Control **(n = 171)**						Intervention **(n = 174)**					
	Substituting protein (5%E)						Substituting protein (5%E)					
	BMI z-score			Overweight status			BMI z-score			Overweight status		
	β	95% CI	P-value	OR	95% CI	P-value	β	95% CI	P-value	OR	95% CI	P-value
**Model 1** [a]												
Carbohydrate	-0.24	-0.44, -0.04	0.02	0.62	0.29, 1.32	0.21	-0.05	-0.26, 0.16	0.64	0.89	0.43, 1.81	0.74
Fat	-0.21	-0.39, -0.02	0.03	0.62	0.31, 1.27	0.20	-0.04	-0.24, 0.16	0.72	0.86	0.42, 1.75	0.67
**Model 2**[b]												
Carbohydrate	-0.28	-0.47, -0.08	0.01	0.53	0.24, 1.18	0.12	-0.05	-0.25, 0.15	0.64	0.82	0.36, 1.89	0.65
Fat	-0.25	-0.44, -0.06	0.01	0.49	0.23, 1.10	0.10	-0.06	-0.25,0.12	0.51	0.72	0.31, 1.67	0.45
	Substituting protein (100 kJ)						Substituting protein (100 kJ)					
	BMI z-score			Overweight status			BMI z-score			Overweight status		
	β	95% CI	P-value	OR	95% CI	P-value	β	95% CI	P-value	OR	95% CI	P-value
**Model 1** [a]												
Carbohydrate	-0.14	-0.25, -0.03	0.01	0.78	0.50, 1.21	0.27	-0.06	-0.19, 0.06	0.33	0.92	0.61, 1.38	0.68

Fat	-0.12	-0.23, -0.01	0.03	0.80	0.53, 1.23	0.32	-0.06	-0.19, 0.08	0.41	0.93	0.59,1.46	0.74
**Model 2** [b]												
Carbohydrate	-0.16	-0.28, -0.05	0.01	0.75	0.47, 1.17	0.20	-0.06	-0.17, 0.06	0.35	0.90	0.58,1.41	0.64
Fat	-0.15	-0.26, -0.04	0.01	0.73	0.46, 1.14	0.17	-0.07	-0.19,0.06	0.29	0.87	0.52,1.45	0.59

[a] Model 1 was adjusted for body mass index (BMI) z-score at 9 months of age.

[b] Model 2 was additionally adjusted for child sex, primary milk source (breastmilk, formula/dairy, or mixed), child birthweight, maternal education, and pre-pregnancy BMI. The sample size (n) for model 2 was 167 for Intervention group and 164 for control group.

Notes: The models represent the iso-energetic increase of 5%E, or a 100 kJ increase from one macronutrient (carbohydrate or fat) with a decrease in the substituting macronutrient (protein).

Supplementary Table S5 Stratified analysis results by intervention allocation for effects of substituting plant and dairy protein for non-dairy animal protein on changes in BMI z-score and overweight status from 9 months to 5 years of age

	Control (n = 171)						Intervention (n = 174)					
	Substituting non-dairy animal protein (5%E)						Substituting non-dairy animal protein (5%E)					
	BMI z-score			Overweight status			BMI z-score			Overweight status		
	β	95% CI	P-value	OR	95% CI	P-value	β	95% CI	P-value	OR	95% CI	P-value
**Model 1**[a]												
Plant protein	-0.02	-0.45,0.41	0.94	2.05	0.34, 12.32	0.43	-0.06	-0.45, 0.32	0.75	1.01	0.28, 3.57	0.99
Dairy protein	0.01	-0.28, 0.31	0.92	1.13	0.32,4.03	0.85	-0.33	-0.69, 0.04	0.08	0.48	0.13, 1.83	0.28
**Model 2** [b]												
Plant protein	0.02	- 0.42, 0.45	0.94	2.63	0.38, 18.33	0.33	0.01	-0.37, 0.34	0.95	2.07	0.46, 9.22	0.34
Dairy protein	0.04	-0.32, 0.40	0.81	0.99	0.21, 4.77	0.99	-0.23	-0.63, 0.17	0.26	0.55	0.09, 3.50	0.53
**Model 3** [c]												
Plant protein	0.06	-0.37, 0.49	0.78	3.39	0.47, 32.29	0.21	0.02	-0.38, 0.34	0.89	1.63	0.35, 7.67	0.54
Dairy protein	0.03	-0.32, 0.39	0.85	1.02	0.21, 5.01	0.98	-0.23	-0.63, 0.17	0.27	0.63	0.09, 4.38	0.65
Substituting non-dairy animal protein (5 g)							Substituting non-dairy animal protein (5 g)					
	BMI z-score			Overweight status			BMI z-score			Overweight status		
	β	95% CI	P-value	OR	95% CI	P-value	β	95% CI	P-value	OR	95% CI	

												P-value
**Model 1** [a]												
Plant protein	-0.06	-0.25, 0.13	0.53	1.11	0.54, 2.33	0.77	-0.01	-0.15,0.13	0.89	1.10	0.70, 1.72	0.69
Dairy protein	-0.02	-0.14, 0.10	0.72	0.91	0.53, 1.53	0.71	-0.14	-0.28, 0.01	0.07	0.86	0.51, 1.46	0.59
**Model 2** [b]												
Plant protein	-0.05	-0.24, 0.14	0.58	1.10	0.51, 2.35	0.81	0.03	-0.11, 0.16	0.69	1.56	0.89, 2.73	0.12
Dairy protein	-0.04	-0.19, 0.11	0.58	0.92	0.51, 1.63	0.76	-0.06	-0.22, 0.10	0.48	1.40	0.64, 3.10	0.40
**Model 3** [c]												
Plant protein	0.05	-0.15, 0.27	0.58	2.12	0.81, 5.56	0.13	0.03	-0.13, 0.18	0.74	1.31	0.71, 2.41	0.39
Dairy protein	0.05	-0.12, 0.21	0.57	1.13	0.54, 2.38	0.73	-0.06	-0.25, 0.13	0.55	1.00	0.39, 2.59	0.99

[a] Model 1 were adjusted for body mass index (BMI) z-score at 9 months of age.

[b] Model 2 was additionally adjusted for child sex, primary milk source (breastmilk, formula/dairy, or mixed), child birthweight, maternal education, and pre-pregnancy BMI.

[c] Model 3 was additionally adjusted for total energy intake at 9 months. The sample size (n) for model 2 and 3 was 167 for Intervention group and 164 for control group.
